# Impact of 12-Month Smartphone Breathing Meditation Program upon Systolic Blood Pressure among Non-Medicated Stage 1 Hypertensive Adults

**DOI:** 10.3390/ijerph17061955

**Published:** 2020-03-17

**Authors:** Jessica Chandler, Luke Sox, Vanessa Diaz, Kinsey Kellam, Allison Neely, Lynne Nemeth, Frank Treiber

**Affiliations:** 1 College of Nursing, Medical University of South Carolina, Charleston, WV 29425, USA; soxl@musc.edu (L.S.); kellam@musc.edu (K.K.); neelyal@musc.edu (A.N.); Nemethl@musc.edu (L.N.); treiberf@musc.edu (F.T.); 2 College of Medicine, Medical University of South Carolina, Charleston, WV 29425, USA; diazva@musc.edu

**Keywords:** hypertension management, meditation, behavior change, blood pressure

## Abstract

(1) Background: Hypertension (HTN) affects ~50% of adults and is a major risk factor for stroke and cardiovascular disease. In 2017, the SPRINT trial outcomes led to lowering of HTN cutoffs by the American College of Cardiology (ACC) and American Heart Association (AHA). The Joint National Committee (JNC8) and National High BP Education Program recommend that lifestyle modifications be used as first-line HTN treatment. Chronic stress is a risk factor for HTN and cardiovascular disease. A recently completed 12 month randomized controlled trial (RCT) of a breathing meditation smart phone app (Tension Tamer, TT) involving JNC8 designated pre-HTN adults provided an opportunity to examine its impact upon individuals now classified as having stage 1 HTN. The TT app captures continuous real-time heart rate (HR) from a user’s fingertip placed over a video camera lens during sessions. Users receive immediate feedback graphs after each session, showing their HR changes. They also receive motivational and social reinforcement SMS text messages the following day based upon levels of adherence. We conducted ancillary analyses of a 2-arm, 12-month, small-scale efficacy RCT among a subgroup of our total sample of participants, who are now classified as having stage 1 non-medicated systolic HTN. Primary outcome was change in resting systolic blood pressure (SBP). Secondary outcomes were change in resting diastolic blood pressure, adherence to the TT protocol, and perceived stress levels. (2) Methods: 30 adults (mean age: 45.0 years; 15 males; 16 White; 14 Black) with ACC/AHA 2017 defined systolic HTN (130–139 mmHg) on 3 consecutive sessions (mean SBP: 132.6 mmHg) were randomly assigned to TT or lifestyle education program delivered via smartphone (SPCTL). Each group received a twice-daily dosage schedule of TT or walking (month 1: 15 min; months 2 and 3: 10 min; months 4–12: 5 min). (3) Results: Mixed modeling results revealed a significant group x time effect for SBP (p<.01). The TT group showed greater SBP reductions at months 3 (−8.0 vs. −1.9), 6 (−10.0 vs. −0.7), and 12: (−11.6 vs. −0.4 mmHg; all *p*-values <0.04). (4) Conclusion: The TT app was beneficial in reducing SBP levels among adults with stage 1 systolic HTN. The TT app may be a promising, scalable first-line tactic for stage 1 HTN. Preparations are underway for an efficacy RCT involving uncontrolled stage 1 HTN patients.

## 1. Introduction

Hypertension (HTN) affects around 49.7% (~55.3 million) of the U.S. population based upon JNC8 guidelines, however the prevalence increases to 63% (~70.1 million) when applying the newly recommended American College of Cardiology/American Heart Association (ACC/AHA) guidelines [1,2]. The 2017 ACC/AHA guidelines recommend using lower systolic blood pressure (SBP) and diastolic BP (DBP) thresholds to define the new classification of stage 1 HTN (SBP: 130 mmHg; DBP: 80 mmHg) [3]. These recommendations are more stringent than the previously used JNC8 guidelines (SBP: 140 mmHg; DBP: 90 mmHg) and have broad implications for the 14.8 million U.S. adults who were previously classified as prehypertensive. 

According to the 2017 ACC/AHA guidelines, those with stage 1 hypertension should first be offered non-pharmacologic, lifestyle modification treatment for 3–6 months prior to initiation of pharmacologic agent(s). The interventions include weight loss for those overweight or obese via heart-healthy diet, including sodium restriction and potassium supplementation, increased physical activity (150 min/week moderate intensity activity) via a structured program, and moderating alcohol intake (men ≤ 2 and women ≤ 1 alcohol drink/day). While there is brief mention of addressing psychosocial stress in the ACC/AHA guidelines for treatment of hypertension, meditation is not formally recommended [3]. The 2018 Canada HTN Guidelines recommend individualized cognitive–behavioral interventions, particularly those that incorporate relaxation techniques for reducing sympathetic arousal [4]. This suggests that while there is not yet long-term proof of sustained benefit of meditation in reducing BP among hypertensives, it is viewed as a possible non-pharmacologic tactic and requires further empirical evaluation to determine potential lasting benefits [5].

Chronic stress (e.g., repeated exposures to discrimination, family dysfunction, marital/relationship discord, financial strain, job strain) has been implicated in development and exacerbation of essential HTN [6,7,8]. A variety of cognitive behavioral stress reduction programs include various forms of meditation [9,10]. Previous randomized controlled trials (RCTs) involving meditation for BP reduction among pre-hypertensives and hypertensives have had mixed results, in part due to high variability in the quality of design methodology [10,11,12]. Many of these RCTs were 1–6 months in duration and involved group, in-person sessions by a certified instructor, followed by home-based practice [10,11,12,13,14].

This intervention delivery approach limits the scalability of meditation programs should they become a recommended treatment option by the ACC/AHA. Importantly, few RCTs objectively monitored non-instructor observed adherence to the prescribed dosage, often relying upon self-report (e.g., journaling, retrospective recall). The true degree of meditation dosage received is unknown, which may partially account for the heterogeneity in participant BP changes, both within and across the various meditation RCTs. 

Our team has been involved in addressing several of these issues by including adults with pre-HTN and HTN in the development of a smartphone-delivered breathing awareness meditation application. The participants preferred the use of brief audio and video clip instructions for demonstrating how to engage in breathing awareness meditation (BAM; diaphragmatic breathing), the first phase of mindfulness meditation [15,16,17,18]. A unique feature included development of proprietary software using reflective photoplethysmography via the phone’s video camera, to enable continuous heart rate acquisition from finger pulsatile flow changes. The heart rate program was validated in comparison to simultaneous ECG recordings with individuals at rest and under acute stress (i.e., videogame challenge, oral presentation) [19]. Following a successful proof of concept study with high acceptability and usability ratings, adherence to twice-daily sessions for 3 months (0.75–1.00 per month) and clinically relevant reductions in resting SBP in pre-hypertensives [20], we conducted a 6-month feasibility trial using the Tension Tamer (TT) smartphone app. The trial sought to determine the optimal dose of BAM comparing three twice-daily dosage schedules (5, 10, and 15 min sessions) upon changes in stress and SBP among JNC8-defined pre-hypertensive adults [21]. Guided by the results from that feasibility trial, we recently completed a small-scale two-arm efficacy RCT to determine BAM’s effects on BP and perceived stress among JNC8 defined pre-hypertensive adults.

The recent change in BP classification guidelines provided the opportunity to examine BAM’s impact upon the 2017 ACC/AHA-defined non-medicated stage 1 hypertensive adults. Therefore, we conducted ancillary analyses of our recently completed 12-month, two-arm, small-scale efficacy RCT (ClinicalTrials.gov identifier: NCT03168789) among a subgroup of adults now classified as having stage 1 HTN based upon the ACC/AHA 2017 guidelines, in order to examine the impact of our BAM program on resting SBP, DBP, and perceived stress [3].

## 2. Materials and Methods

### 2.1. Research Design

This investigation was an ancillary analyses of a two-arm, 12-month, small-scale efficacy randomized controlled study (RCT) among a subgroup of adults now classified as having stage 1 non-medicated systolic hypertension. Out of 388 screened potential participants, we enrolled 84 into the original RCT. Of those 84 participants, data from 30 participants now classified as having stage 1 HTN were included in the current analysis. As part of the main RCT, there were two groups: (1) an experimental group (TT) and (2) a lifestyle education program delivered via smartphone (SPCTL) group. The Institutional Research Board of the Medical University of South Carolina approved the study (Pro00020894). All participants provided written informed consent. The study spanned from November 2016 to November 2018.

### 2.2. Participants

The study team screened 388 adults using flyers and clinic referrals for pre-HTN in Charleston, SC, from November 2016 through November 2018. Inclusion criteria consisted of: non-Hispanic White or African American men or women, 18–90 years old, and systolic pre-HTN based upon the previously utilized JNC8 guidelines (121–139 mmHg) on 3 consecutive visits, each separated by at least 2 days, with the third visit comprising the full baseline evaluation, including an average of the three visits’ blood pressure results as the baseline blood pressure value. Exclusion criteria consisted of any chronic illness or medical condition requiring regular pharmacological intervention that may affect BP; unwillingness to be randomized into one of two study arms (TT or SPCTL); inability to use a smartphone (e.g., open and navigate the TT or Runkeeper smartphone applications after hands-on training; inability to position finger over smartphone camera; or inability to see the feedback graphs or listen to audio and video clip instructions on the app); participating in another research study of any kind; inability to speak, hear, or understand English; or pregnant, lactating, or intention of becoming pregnant during the trial.

**Figure 1:**
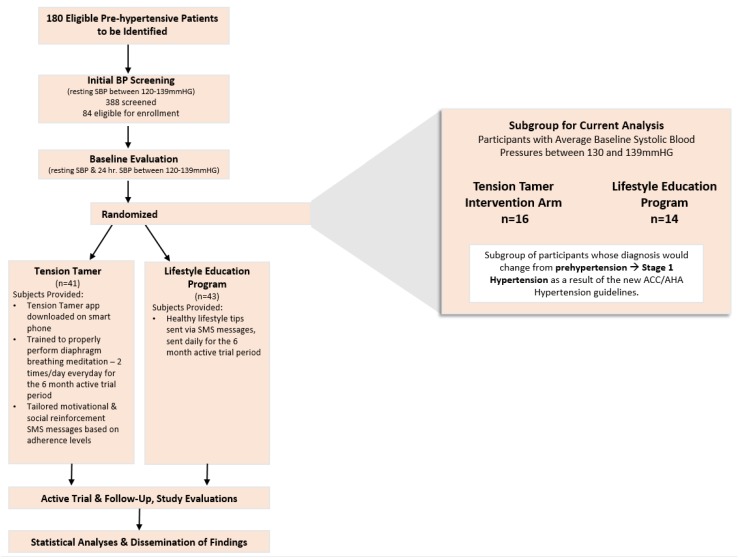
Consort diagram for tension tamer RCT.

Research staff contacted interested participants via telephone to determine interest and schedule the first of three potential BP screening visits. Details of the BP protocol used at the BP screenings and subsequent clinical evaluations are published elsewhere [21]. Of the 388 individuals screened, 306 were found to be ineligible due to SBP being below the pre-HTN range during one of the three evaluations. The remaining 84 consented and enrolled into the RCT. Of the 84 participants in the initial study, 30 participants’ data were included in the present analysis (see Figure 1 for CONSORT diagram). 

### 2.3. Protocol, Intervention, and Groups

#### 2.3.1. Tension Tamer (TT) Intervention Group

TT is a patient-centered, iteratively designed breathing awareness meditation mobile application (app) compatible with Android or iOS platforms. The TT app’s content and implementation formats were guided by underlying tenants of self-determination and social cognitive theories [22,23]. Collectively, these theories maintain that one needs to develop self-efficacy, competence, and autonomous regulation (i.e., intrinsic motivation) for the desired behavior changes to initiate and sustain over time. The people, activity, context, and technology approach (PACT) model also guided TT app development and posits that users must feel at ease in using the technology and perceive its usefulness in helping them reach a desired goal [24]. It is postulated that with positive feedback and social reinforcement (e.g., TT app’s immediate post-session heart rate feedback charts, tailored motivational and social reinforcement text messages based upon levels of adherence), self-efficacy and autonomous regulation will increase, contributing to consistent utilization and sustained engagement over time. An example message that a TT participant would receive might read: *“You completed two Tension Tamer sessions over the last 24 h! Remember, the more you practice, the more it becomes a part of your routine.”*

The TT app utilizes a smartphone’s camera lens to acquire continuous measures of heart rate via detection of fingertip pulsatile blood flow changes via proprietary reflective photoplethysmography software. The date and time stamp information of heart rate acquisition across the session is a proxy for adherence. The participant must keep the tip of their index finger on the camera lens during meditation sessions. Within 20–30 s, the app acquires a stable heart rate and continuously displays heart rate presented on a 4 beat rolling average. TT-app-derived heart rate has been validated against ECG at rest and during acute stress exposure (i.e., oral presentation, videogame challenge) [19]. Additionally, there is a video clip guide, in which a moderator describes how to engage in BAM (diaphragmatic breathing, also known as belly breathing), while showing an individual engaging in BAM with attention to the expansion of the abdominal region when inhaling through the nose. There is also an audio guide, which automatically plays on the initial BAM session. A female with a British accent leads participants through the BAM session, including directions on finding a quiet, comfortable location to sit comfortably or lie down, activate the TT heart rate acquisition component, and engage in breathing in a slow, deep, relaxed manner, while attending to the sensations of one’s breathing. She also provides guidance on how to regain attention to one’s breathing when distractions occur (e.g., external noises, such as people talking, car horn blaring, intrusion of random thoughts, worries, etc.). After the initial session, users may elect to deactivate the audio guide on future sessions if desired.

A brief chime activates midway and at completion of the session. The TT app provides a timer on the screen, which displays the duration of each session. If there is 30 s of excessive movement of the phone preventing heart rate acquisition, the phone will vibrate and display a message on the app screen requesting less movement and to refocus upon engaging in the meditation session. If a participant has more than two disruptions during the session, the TT session halts and a message is presented requesting the participant to complete a session when they find a better time to engage in BAM. The TT app includes a technology assistance phone number button should users require any assistance in the future.

**Figure 2:**
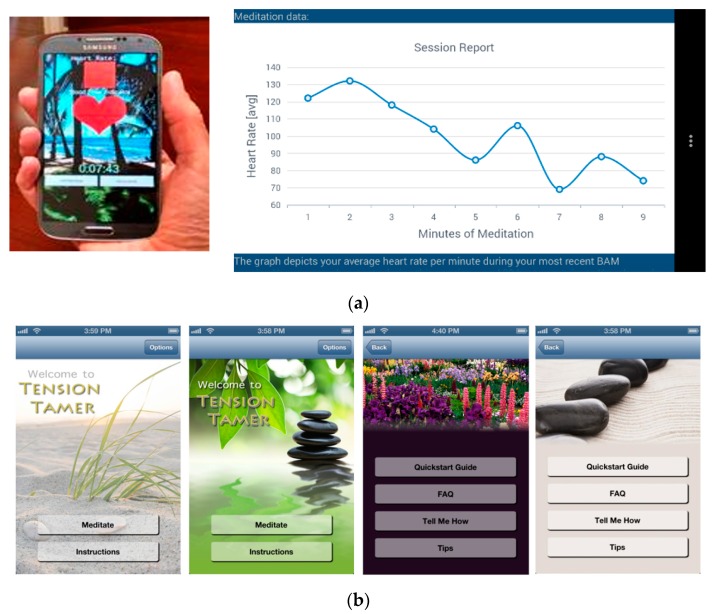
(**a**) Depiction of in-app heart rate acquisition and feedback graph; (**b**) Examples of setting options of the tension tamer mobile app.

Immediately following completion of a TT session, users receive a feedback graph displaying average heart rate each minute and maximum decrease observed (see Figure 2a). Users have the option to turn off (or reactivate) the audio BAM instructions, set middle or end of session alerts (chime, gong), and select different background themes of screenshots (e.g., nature, rock garden, beach scenes—see Figure 2b). The TT app sends encrypted heart rate data to our institution’s HIPAA-compliant relational database management system via a securely authenticated Web API. 

During the baseline visit following informed consent and randomization, a trained team member downloaded the TT app to the participant’s phone and instructed them in navigating each module (e.g., explained BAM, activating and deactivating the audio instruction guide, frequently asked questions, accessing feedback graphs, technology assistance phone line, etc.). Each participant was given additional guidance and feedback as needed, while practicing BAM and listening to the audio instructions to acquire their heart rate through using the app. When comfortable, they completed their first complete BAM session on their own using the audio instruction guide. After viewing the immediate post-session summary graph, participants had the opportunity to ask any questions they may have had.

Based upon results from our previous dose–response trial [21], each TT participant was instructed to complete two 15-min daily sessions for the first month, decrease to two 10-min daily sessions for months 2 and 3, and then decrease to 5-min sessions for the remainder of the 12-month trial. If desired, one could engage in more than the 2 sessions/day dosage, however our analysis only included adherence to the prescribed dosage, which was determined to be most efficacious in a previous trial [21]. For example, if a participant engaged in an extra session each day (i.e., 3 sessions instead of the prescribed 2), they received the same adherence score as a participant who completed the prescribed dosage, with each receiving a score of 1.0. 

#### 2.3.2. Lifestyle Education Program Delivered via Smartphone (SPCTL)

The SPCTL attention control group received the same twice-daily dosage schedule for engagement in a walking or running program using the Runkeeper^TM^ app (month 1: 15 min sessions; months 2 and 3: 10 min sessions; months 4–12: 5 min sessions). As with the TT group, if they wished to engage in longer or more frequent daily sessions, this was permitted. A research assistant downloaded the Runkeeper^TM^ app to each SPCTL participant’s phone, demonstrated all features of the app, and ensured the participants’ understanding of the app. The app was used to enable tracking and provide feedback of the duration of daily planned physical activities (e.g., outdoor walk or run, treadmill, strength exercises, etc.) participants performed the amount of total daily activity using the phone’s built in accelerometer (steps/day) and GPS software for distance tracking.

The SPCTL group also received the same frequency of SMS messages as the TT group, however content did not pertain to their level of regimen adherence. They received healthy lifestyle-behavior-related educational messages associated with their heart-healthy diet, low sodium intake, non-smoking, physical activity, sun exposure, and other factors. An example message that a SPCTL participant would receive might read: *“Positive mental health allows people to reach their full potential, cope better with stress, and make meaningful contributions to their communities.”* As they progressed in the trial, similar to the TT group, participants could immediately access cumulative charts showing their amount of steps/day and duration of exercise episodes per week and month, as well as their adherence levels to their dosage schedules across the 12 months.

### 2.4. Outcome Measures

The primary outcome measure was resting SBP levels. Secondary outcomes included SBP control and adherence to the Tension Tamer and Runkeeper protocols. Secondary outcomes were DBP control, changes in resting DBP, and perceived stress as measured by the perceived stress scale [25]. 

### 2.5. Statistical Analyses

Analyses were completed in December 2019. Prior to analyses of primary outcomes, descriptive means and standard deviations (SD) of participants, including age, race, education level, marital status, income, and employment, were examined and reported. For relevant categorical measures (e.g., meeting adherence benchmarks versus not), chi-square tests were used to compare between groups. Primary outcome measures are changes from pre-intervention in resting SBP to resting SBP, measured at 3, 6, and 12 months. Simple unadjusted mean change in resting SBP from pre-trial to 3, 6, and 12 months (between groups) were compared using Student’s t-tests. SBP changes were compared for the 2 groups (i.e., TT vs. SPCTL) using a GLM approach with mean resting SBP values as the dependent variable, with a pre-calculated and significant time by group interaction term added to the model. All analyses were performed using STATA (v.14.0, College Station, TX, USA).

## 3. Results

### 3.1. Recruitment and Retention Rates 

Of a total of 388 screening participants, we enrolled 84 into the original RCT. Of those 84, based upon their average SBP across the three BP screenings, 30 participants classified as stage 1 HTN according to the 2017 ACC/AHA Guidelines. They were selected for this study. We observed an overall recruitment rate of 100% (84/84). With regard to overall retention rate, 62 (75.5%) completed the 12 month trial (31 TT participants, 31 SPCTL participants). Two participants (one from each group) were dropped when they became ineligible due to being diagnosed with chronic diseases involving medication, which can effect BP (i.e., cancer and Parkinson’s, hypertension). Fourteen (6 TTs, 8 SPCTL s) lost interest during the first 1–1.5 months and dropped out, while 2 TTs withdrew during month 7, citing time commitment issues (unable to make laboratory evaluations, one moved out of state). 

The 12-month retention rate for the subgroup of 30 participants classified as having stage one HTN (SBP ≥ 130 mmHg) was 86.7% (26/30). One SPCTL dropped out due to loss of interest in month 7 and three TTs dropped out between months 2–7 due to either loss of interest or moving out of the area.

Finally, similar to the entire cohort, there were no statistically significant differences between this study’s TT and SPCTL subgroups across any of the demographic or baseline descriptive variables presented in Table 1 (all *p* values > 0.70 using Student’s T-test).

### 3.2. Blood Pressure Control Changes 

A primary outcome variable was the change in SBP control (i.e., <130 mmHg). As expected and shown in Table 2, at baseline evaluation no subject met the 2017 ACC/AHA SBP control cut off (<130 mmHg). Using Fischer’s exact test, significantly higher percentages of SBP control were demonstrated by the TT group at months 6 and 12 compared to the HLAC group (all *p*-values < 0.02). While not statistically significant, a higher proportion of the TT group achieved SBP control at the other two earlier evaluations (months 1 and 3). Lastly, compared to the SPCTL group, the TT group exhibited higher % of SBP control (<130 mmHg) across the duration of the active trial (60.3% vs. 35.8%; *p* = 0.003). 

Since uncontrolled DBP was not used as an inclusion criteria, there were individuals in each group that had controlled DBP. As shown in Table 3, 55% of TT participants and 60% of SPCTL participants exhibited controlled DBP at baseline. There were no significant differences in proportion of controlled DBP based upon a chi-square analysis (X^2^ (1, *n* = 30) = 1.07, *p* = 0.301). During the course of the trial, both groups showed similar increases in DBP control, resulting in non-statistically significant differences between the groups (all *p*-values > 0.35).

### 3.3. Resting Blood Pressure Level Changes 

**Figure 3:**
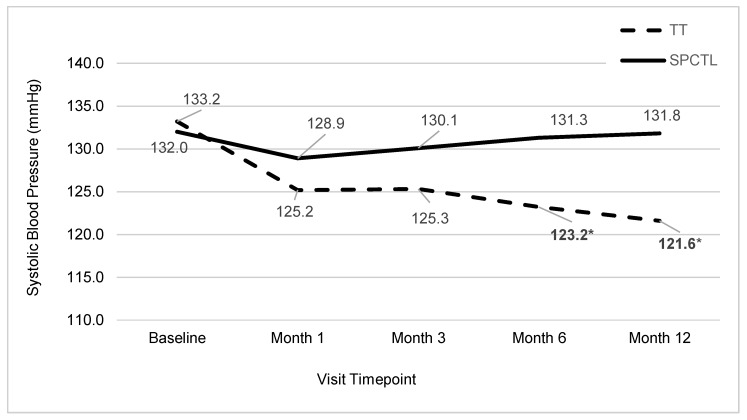
Average Systolic Blood Pressure by Group and Visit.

The other primary BP outcome variable was the change in SBP levels. There were no statistically significant differences between the TT and SPCTL groups in resting SBP at baseline (*p* = 0.12). General linear mixed model (GLMM) analyses of treatment over time (BL, 1, 3, 6, 12 months) revealed a significant treatment by time interaction for SBP (Figure 3, *p* < 0.001).

Post hoc analyses indicated that the TT group showed greater SBP reductions at months 3 (−7.9 vs. −1.9), 6 (−10.0 vs. −0.9), and 12: (−11.6 vs. −0.4 mmHg; 6 and 12 month *p*-values < 0.04). 

**Figure 4:**
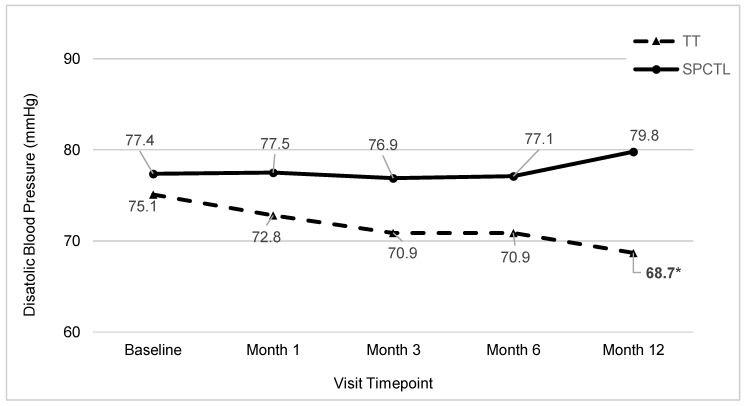
Average Diastolic Blood Pressure by Group and Visit.

As with the resting SBP findings, the TT and SPCTL groups were not significantly different at baseline for resting DBP (*p* > 0.45). The GLMM analyses indicated a significant treatment by time interaction for DBP (*p* = 0.01). Post hoc analysis indicated that at the 3, 6, and 12 month time points, DBP levels were significantly lower in the TT group (Figure 4, all *p*-values < 0.01).

### 3.4. Adherence to TT and Runkeeper Dosage Rates 

As in our earlier BAM feasibility trials including several involving the TT app, adherence was defined as completion of 2 entire sessions per day [19,20,21]. Partial adherence (0.5) was allotted for completion of one of two assigned sessions for the day. As noted earlier, for TT, encrypted time and date stamped heart rate data measured via the app’s built-in photo plethysmograph were automatically relayed to the institution’s server infrastructure [19]. These data were used as objective measures of adherence (i.e., 1.0 = 2 TT sessions at full dosage over a 24 h period, with greater than five minutes separation between sessions). Adherence was reported on a monthly percentage calculated as average of daily adherence scores (0, 0.5, or 1.0) across a number of days in the month. Adherence categories were defined as <75% or >/= 75% based on our preliminary studies and other previous meditation RCTs, where 75% adherence was associated with clinically meaningful BP reductions [9,10,21,26].

Those in the SPCTL group had adherence scores derived from their Runkeeper app’s daily output of the number and duration per session of physical activity for the previous 24 h. Scores were calculated in the same manner as the TT adherence scores. Mean monthly adherence measures by group are shown in Table 4. Comparisons of mean by group and study month were achieved using GLM modeling analysis. The proportion of participants meeting at least 75% of sessions are shown in Table 5. Adherence was measured by calculating percentage of completed sessions divided by the expected sessions for the study month. The data captured by the TT or Runkeeper app was uploaded to the institution’s servers. The *p* values were calculated using chi-square tests. 

### 3.5. Perceived Stress

Perceived stress classifications by group and timepoint are shown in Table 6. There were no significant differences between or within groups at any timepoints.

## 4. Discussion

The 2017 ACC/AHA and Canada HTN Guidelines recommend that patients with stage 1 HTN should first be offered non-pharmacologic, lifestyle modification treatment prior to initiation of pharmacologic agent(s). Although not officially a recommendation due to scarce evidence of long lasting effects, the guidelines discuss meditation as a means of addressing psychosocial stress for treatment of stage 1 HTN. This ancillary analysis of our recently completed 12 month efficacy RCT examined the effects of breathing awareness mediation (BAM) among a subgroup of adults now classified as having stage 1 HTN based upon the ACC/AHA 2017 guidelines. 

The current findings corroborate and extend previous meditation RCTs that involved adolescents and adults with pre-HTN or HTN (SBP > 130 mmHg). The TT app group demonstrated increasing average SBP reductions across the trial from baseline levels, ranging from 8.0 mmHg at the first month to 11.6 mmHg at the final 12 month evaluation. Our TT group findings corroborate and extend earlier meditation RCT findings. A meta-analysis of 25 RCTs evaluated the impacts of various breathing relaxation tactics, including BAM, which were often delivered as part of mindfulness meditation programs among adult hypertensives (SBP ≥ 140 mmHg). Hypertensives experienced an average mean reduction difference of 5.5 mmHg SBP compared to attention control groups across the various trials, which averaged 3 months duration [27]. Several RCTs that collectively involved adults with pre-HTN and HTN found that BAM administered 10 minutes twice daily for three months resulted in average SBP reductions, ranging from 4.3 to 7.2 mmHg [28,29]. 

Our findings revealed a comparable mean difference in SBP reduction of 7.9 mmHg at month 3 compared to the attention control physical activity program. Importantly, our RCT is one of the longer meditation trials, with a duration of 12 months. Our results suggest that individuals with HTN can sustain engagement in BAM for 12 months and experience further reductions in resting SBP (10 to 11.6 mmHg at 6 and 12 months, respectively). These reductions in BP are quite respectable compared to other lifestyle interventions for BP control among hypertensives; that is, typical lifestyle weight loss interventions with hypertensives have shown 4–5 mmHg decrease in SBP and 2–4 mmHg decrease in DBP [30,31,32]. The combination of a heart-healthy diet and low sodium intake may decrease SBP by about 11 mmHg [3]. 

Participants’ adherence to the TT app was not significantly different from the enhanced attention control group’s adherence to the Runkeeper app for recording engagement in physical activity; however, the TT group experienced SBP decreases of greater magnitude and sustained changes through the 12 month visit. The current analysis revealed clinically meaningful reductions and statistically significant reductions in SBP by the 6 and 12 month evaluation for the TT group, illustrating the value of BAM as a first line treatment for stage 1 HTN for those patients willing to partake. Anecdotally, key informant interviews conducted at the end of the 12 month trial suggested that many of those who were still active meditators began practicing BAM by month 6, without using their phone to acquire their heart rate. They noted they would use the technique when experiencing various feelings of anxiety, tension, and frustration. 

A recent review by Goyal 2014 et al. [33] reported on 42 meditation studies and their ability to improve stress-related outcomes in adult populations. Of the 42 studies, 19 assessed the effects on psychological stress or distress, with a combined null effect. While there was inconclusive evidence to conclude the effect of meditation programs on psychological distress, it should be noted that on average, the meditation programs lasted for 8.9 weeks (i.e., just over two months). A review article by Davidson and Kozniak [18] noted that meditation programs with defined outcomes, such as perceived stress, hostility, anger, and negative effects, should employ a greater trial duration and more frequent follow-up evaluations to determine lasting effects. The authors noted that shorter-term studies might only experience transient and short-term effects, while longer-term trials may result in increased attrition but lasting results for adherent participants. Consistent with these results of null findings, the proportion of participants in this analysis classified as “high stress” across the trial was not significantly different in the TT meditation group compared to the SPCTL group. Although not statistically significant, the trend towards improvements in perceived stress gives rationale towards an examination into the effects of the fully powered trial, including all 84 participants, possibly indicating that longer term engagement in meditation may be necessary for significant improvements in perceived stress or psychological distress. 

Several unique aspects included in our methodology addressed common weaknesses observed in the majority of the earlier meditation trials. The use of a smartphone application to deliver and monitor participants’ engagement in sessions is perhaps the most unique contribution. Capitalizing upon the phone’s video camera, reflective photoplethysmography was incorporated to acquire participants’ continuous heart rates to serve as a proxy for adherence. It also enabled provision of immediate feedback to the user of how their heart rate was declining during the session, as well as a summary graph depicting the pattern of heart rate change across the session. The cumulative charting file enabled comparison of average session heart rates across weeks and months. Adherence to meditation programs has been cited as one likely reason for the wide disparity in outcomes both within and across various types of meditation trials [9,10,18,33]. Typically, adherence outside of direct observation during training sessions has relied upon self-report, either through journaling or retrospective recall. Another distinct methodological feature of our TT app is the embedded audio and video guide tutorials for participants to refer to when assistance with breathing awareness meditation was desired. This feature allowed participants to partake in on-demand retraining of BAM as required. Finally, the 12 month duration of our RCT addresses one of the primary weaknesses of earlier trials, with most running from 1 to 3 months.

Psychological distress is a major risk factor for the development or exacerbation of hypertension, therefore targeting affective distress, anxiety, depression, and anger or hostility with coping strategies, including meditation, may have a preventative or treatment effect on blood pressure [34]. The current 2017 ACC/AHA Guidelines’ exclusion of meditation as a nonpharmacologic strategy for blood pressure treatment is based upon a review of literature including studies completed prior to 2014, resulting in a recently penned letter to the editor, expressing the authors’ suggestion to update its reviews and recommendations on non-pharmacologic interventions for BP. While our findings support the continued investigation into the effects of meditation as a first line of treatment for stage 1 HTN, this study does have limitations that should be considered when interpreting the results. First, the small sample size hinders the generalizability of the findings. Second, the study design did not account for levels of physical activity that the meditation group engaged in, therefore causation of SBP reductions cannot be definitively explained. Third, the SPCTL group did not record or indicate which types of physical activities they performed, therefore we cannot generalize that using any physical activity program yields poor results as compared to our mediation program. Finally, the study design (i.e., a post hoc ancillary analysis) limits the ability to infer definitive conclusions; however, it does provide a basis for further empirical scrutiny.

## 5. Conclusions

In conclusion, our findings to this analysis of a breathing awareness meditation program delivered via a health app suggest that a properly powered trial may be warranted to better determine BAM’s effects on blood pressure control in stage 1 hypertensive adults. With our small sample size and long trial period, we were able to demonstrate feasibility of acceptable adherence to a twice-daily meditation program and preliminary lasting effects on blood pressure reductions. With the end goal of being formally recognized as a first line approach to hypertension treatment, breathing awareness meditation’s preliminary effects on SBP control appear promising with proper practice techniques and high levels of adherence.

## Figures and Tables

**Table 1:** Descriptive characteristics of participants (*n* = 30). TT, Tension Tamer; SPCTL, lifestyle education program delivered via smartphone.

Demographic	Self-Reported Response	TT (*n* = 16)	SPCTL (*n* = 14)
Age (M+/− SD)		46.5 ± 13.0	43.4 ± 14.2
Gender	Male	48.7%	49.3%
	Female	51.3%	50.7%
Race	African American	51.3%	42.1%
	White	48.7%	57.9%
Marital Status	Single	6.7%	20%
	Married/Living with Significant Other	73.3%	66.7%
	Separated/Divorced	13.3%	6.7%
	Widowed	6.7%	6.7%
Education	High School or less	6.7%	6.7%
	Partial/College Grad	93.3%	93.3%
Income	$0–25,000	20.0%	33.3%
	$25–50,000	13.3%	13.3%
	>$50,000	53.3%	53.3%
	Not Reported	13.3%	0%
Employment	Full Time	66.7%	73.3%
	Part-Time	13.3%	0%
	Retired/Disabled	20.0%	26.6%

M: mean; SD: standard deviation; *n* = sample size; TT: Tension Tamer group; SPCTL: lifestyle education program delivered via smartphone group; $: USD.

**Table 2:** Proportion of participants meeting the 2017 ACC/AHA Revised Guidelines for Controlled SBP (<130 mmHg).

Controlled SBP	TT	SPCTL	*p*-Value
Baseline	0	0	0.481
Month 1	68.8	35.7	0.074
Month 3	73.3	57.1	0.377
Month 6	78.6	35.7	**0.021**
Month 12	91.7	50.0	**0.029**

Bold Text indicates significance at the *p* ≤ 0.05 level. SBP: systolic blood pressure; TT: Tension Tamer group; SPCTL: lifestyle education program delivered via smartphone group.

**Table 3:** Proportion of participants meeting the 2017 ACC/AHA Revised Guidelines for Controlled DBP (<80 mmHg).

Controlled DBP	TT	SPCTL	*p*-Value
Baseline	Baseline	75.0	0.771
Month 1	Month 1	87.5	0.642
Month 3	Month 3	86.7	0.642
Month 6	Month 6	85.7	0.571
Month 9	Month 12	83.3	0.600

DBP: diastolic blood pressure; TT: Tension Tamer group; SPCTL: lifestyle education program delivered via smartphone group.

**Table 4:** Mean Adherence to protocol by group and timepoint.

Time-Point	TT	SPCTL	*p*-Value
Month 1	77.3	68.2	0.288
Month 3	77.1	67.1	0.408
Month 6	78.0	61.5	0.189
Month 12	69.8	65.2	0.760

TT: Tension Tamer group; SPCTL: lifestyle education program delivered via smartphone group.

**Table 5:** Proportion of participants meeting 75% adherence benchmark.

Timepoint	TT	HLAC	*p*-Value
Month 1	60.0	50.0	0.604
Month 3	64.3	57.1	0.712
Month 6	58.3	35.7	0.267
Month 12	38.5	27.3	0.582

TT: Tension Tamer group; SPCTL: lifestyle education program delivered via smartphone group.

**Table 6:** Proportion of participants categorized as highly stressed based upon the perceived stress scale.

Timepoint	TT	SPCTL	*p*-Value
Baseline	56.3	64.2	0.667
Month 1	56.3	60.8	0.182
Month 3	50.0	42.9	0.717
Month 6	23.1	46.2	0.233
Month 12	38.5	50.0	0.600

TT: Tension Tamer group; SPCTL: lifestyle education program delivered via smartphone group.
